# Examining active aging among Iranian provinces: a TOPSIS analysis

**DOI:** 10.1186/s12889-022-13040-z

**Published:** 2022-04-15

**Authors:** Vahid Saberzadeh, Sara Emamgholipour Sefiddashti, Majid Safaei Lari

**Affiliations:** Present Address: Department of Health Management and Economics, School of Public Health, Tehran University of Medical Sciences, Tehran, Iran

**Keywords:** Active aging, Provincial planning, Poverty rate, Relative welfare, Economic participation rate, Life expectancy

## Abstract

**Background:**

Planning and decision-making for the elderly requires a special attention due to the beginning of aging process in Iran. By emphasizing the concept of active aging, determining the status of the elderly like their ability to continue work over time, to attain income and to participate in social and political life is significant. Active aging uses the indicators measuring the non-used potential of the elderly for having an active and healthy aging. This study aimed to determine the level of active aging among the provinces of Iran in 2018 by considering 11 indicators related to elderly’s health, well-being and socioeconomic participation.

**Methods:**

The raw data were obtained from Statistical Center of Iran. After establishing the indicators based on the Global Age Watch approach, the provinces were ranked by the TOPSIS method in terms of aging status.

**Results:**

The results indicated that only Tehran and Alborz had the highest development level of active aging while 16% of the provinces had a semi-developed status and 77% failed at experiencing a satisfactory welfare, economic and social status.

**Conclusions:**

Four indicators had the highest importance included the percentage of the elderly with a diploma and academic degree, the percentage of the elderly with lower incomes than the median income, the median income of the elderly to the median income of the other people in society, and life expectancy among the 65-year old men. Studying the indicators deeply can result in the appropriate planning for each area in line with the improvement of the elderly status.

## Background

The reduction of fertility rates and increase in life expectancy have led to population aging during the last century [1, 2]. Based on the World Health Organization’s (WHO) report, global life expectancy was 72 years during 2015–2020, which is estimated to be 77 during 2045–2050 [3]. Worldwide, there were 703 million individuals aged 65 years or more in 2019. The number of older persons is estimated to double to 1.5 billion by 2050. Globally, the share of the population aged 65 years or over increased from 6% in 1990 to 9% in 2019. That proportion is projected to rise further to 16% by 2050, so that one in six people in the world will be aged 65 years or over [4].

Based on the definition by the World Health Organization, the people aged 60 or older are considered to be elderly. In addition, that population will be considered elderly if the ratio of the population over 60 is more than 10% of the total population [5].

Population aging should be considered as a success in human beings because the product is a process of long-lasting adaptation. In other words, life expectancy increased through the development of lifelong education, biomedical advances, socioeconomic progress, and political expansion. However, the number has been doubled in some countries over a century [6].

A new paradigm was conducted with a positive look at aging research from the evidence-based point of view during the last decades of twentieth century. This positive aging view approved several verbal words such as healthy [6], successful [7, 8], optimal [9], vital [10], productive [11], active [12], positive [13] or simply good aging [14] or good life. Butler confirmed the study conducted by MacArthur about aging indicating that interaction with meaningful activities leads to health, life satisfaction, and longevity [15]. In addition, the elderly can be effective in society as an economic source. Punyakaew et al. performed a study to examine the active ageing level of healthy elderly people in north of Thailand which showed active ageing was at the moderate level and elderly people at the high level of active ageing engaged in leisure and social participation more than those lower levels [16]. This is while a study by Haque et al. also revealed active ageing level of elderly yet to be improved in Thailand as active ageing level in Thailand is not high (evidenced as far behind the goal). For increasing active ageing level of elderly in Thailand policy should be focused for elderly to fulfill health needs, to promote longer working lives, to arrange lifelong a learning program, and to improve financial/economic conditions [17]. Another survey in Brazil demonstrates that access to public spaces for the practice of physical activity near the home and higher level of education, income, physical health and mental health were more prevalent among older adults with a high level of active aging [18].

Active aging means continuous participation in social, economic, cultural, spiritual, civil, and physical affairs [15]. The concept of active aging developed in the 1990s with an emphasis on the relationship between activity and health. Active aging aims to facilitate the rights of the elderly to stay healthy (reducing the cost of health and social care), continue working overtime (reducing retirement expenses), and participate in social and political life [19].

Due to the increasing aging population in all countries, the process of the onset of active aging, according to the desired indicators, has been faster in developed countries than in other countries [20]. In addition, this phenomenon has started in less developed countries during the recent decades indicating its universality [6].

In order to explain active aging, the indicators measuring the non-used potential of the elderly for having an active and healthy aging were used. Such indicators determine the level of independent living of the elderly, participation in the labor force and social activities in terms of their capacity having four dimensions of employment, participation in society, healthy and independent life, capacity and capable environment for active aging. Each of these dimensions has their own specific indicators specifying the extent of each dimension [21].

HelpAge International NGO developed a multi-dimensional benchmark for the health and well-being of the elderly in 2012. First, aging was studied in the twenty-first century and then indicated the need to monitor the progress of the government in eradicating poverty, improving health, and promoting the participation of the elderly. The Global Age Watch Index was introduced by the HelpAge International in 2013 and had been published annually by 2015. This indicator was an effective advertising tool and highlighted key gaps in aging and the elderly worldwide [22].

Income security is one of the most significant concerns of the elderly throughout the world. Income-related issues with the health of the elderly are among the biggest challenges for governments facing aging populations. The global economic crisis has increased financial pressure to ensure economic security and access to healthcare for the elderly. In order to realize their right to enjoy the highest levels of physical and mental health, the elderly should have access to affordable healthcare and information for satisfying their needs including the preventive, therapeutic, and long-term care. Furthermore, friendly physical environments help develop and use the innovative technologies supporting the active aging especially when people become older and experience less mobility as well as visual and hearing impairment [23]. The indicators of this benchmark are based on the introduction of the International HelpAge Institute including [14] income security {coverage of pension income measuring the presence and coverage of pension scheme in the country, poverty rate in the elderly dealing with the poverty of the elderly using the definition of relative poverty, relative welfare of the elderly measuring the income / consumption status of the elderly relative to the rest of the population, per capita gross national income studying whether all citizens, old and young, equally benefit from increasing economic production in the country}, Health status {life expectancy at the age of 60 measuring how long a 60-year-old person can expect to live, healthy life expectancy at the age of 60 measuring how long a 60-year-old person can expect to live a healthy life, mental well-being measuring the self-assessed mental health by the person and completing the healthy life expectancy indicator, which only relies on physical health. The percentage of people 50 years old and older having a meaningful life than the 35–49 years old people having the same feeling}, personal capability {participation in the elderly labor market (employment rate) reviewing the measures taken by the elderly to access the labor market (both formal and informal employment), and their capability to add pension income to their wages and access to job-supporting networks. Thus, employment rate is used as a proxy for the economic empowerment of the elderly, educational achievements of the elderly examining key competencies in the form of knowledge, skills, and attitudes, and improved quality of life at an older age. Education is a proxy for the lifelong learning of the skills and abilities indicating the potential of social and human capital of the elderly}, empowering the society and environment (participation in society) {social communication measuring the support by relatives and friends, physical security indicating how people feel safe in their neighborhood, civil liberty examining the level of control that the elderly feel about their lives, access to public transportation examining the availability and quality of public transportation which is a key factor in the quality of life of the elderly and gives them access to services (such as health care and shops), friends, and family}.

The increase of aging population is one of the most important economic, social and health challenges in the twenty-first century [24]. Because this event varies in different countries, it can create social, economic and cultural challenges at different levels of individual, family and social in all countries [1] and effects on productivity and economic growth [25]. Therefore, in order to hinder unfavorable consequences of aging population, active aging should be promoted.

Despite the fact that there are corpus of knowledge and many researches on active aging, it is preliminarily required to assess the existent situation and levels of active aging in society. Henceforth, policy makers are able to make appropriate decisions and policies for promoting active aging. Thereupon, this study aimed to investigate the active and healthy aging status of the elderly in the provinces of Iran by considering that the process of aging has begun in Iran according to demographic and statistical indicators [26] and because no comprehensive study was conducted on the welfare state of the elderly in Iran and there is no information for planning this group of people.

## Methods

The present study was descriptive in terms of method and cross-sectional in terms of time. This study aimed to determine the level of active aging in the provinces of Iran in 2018 by considering 11 indicators related to the elderly including their health, well-being, and participation. In order to collect data, the information related to two parts were used. One part was the information of people over 65 years old at the last census of 2016 collecting from the website of Statistical Center of Iran and using the population and housing census results. Then, the results were entered into Excel software. The indicators elicited from the information included special economic participation rate for people over 65 years (the access of the elderly to the labor market), ratio of the households with a householder over 65 (social communication), people over the age of 65 with diploma and higher academic degrees (educational access), the elderly over 65 living in the households with more than one member (independent living), life expectancy at 65-year-old men and women (health status), and the elderly over 65 (social communication). The second part included the information related all people over 65 in the household income survey in 2018. The required indicators including the percentage of the people over 65 receiving salary (salary and pension coverage), percentage of the people over 65 with a lower income than the middle income of the country (the poverty rate of the elderly), and the median income of the people over 65 to the median income of the rest of society (relative welfare) were obtained by referring to Statistical Center of Iran website, obtaining the results of the household income census, sorting the Access file, and then importing the data into Excel.

### Measuring the indicators in each dimension

- In terms of income security, the percentage of people with lower income than the middle income of the country (the poverty rate of the elderly), median income of the people over 65 to the median income of the rest of the population (relative welfare), and the percentage of the people over 65 years receiving salary (salary and pension coverage) were used. All these indicators were obtained from household income data and the poverty rate of the elderly, their relative welfare, and the amount of their salary and pension coverage were examined. The above-mentioned indicators were measured as follows:
  $$\frac{People\ over\ 65\ years\ of\ age\ have\ a\ lower\ income\ than\ middle \ income* 100\ }{Population\ over\ 65\ years\ of\ the\ province}=\mathrm{Elderly}\ \mathrm{Poverty}\ \mathrm{Rate}$$

In order to measure the above-mentioned indicators, all people having lower incomes than the median total income of the country were calculated and divided into the sampled population of the province. In order to measure the median income of the whole country, the total income of a rural and urban household was identified separately by referring to the results of the rural-urban household income survey. Then, each urban and rural income was separately divided by the mean square root of the number of people in the household. In addition, the income of each person above the age 65 years was divided by the square root of that person’s household size. Finally, the median income of the country for urban and rural areas equated with household size was compared to the equivalent income of the urban and rural elderly in each province and the individuals in each region (urban or rural) were compared based on the median income of their region.

$$\frac{Average\ income\ for\ people\ over\ 65\ in\ each\ province\ }{Middle\ income\ of\ the\ whole\ country}=\mathrm{Relative}\ \mathrm{Welfare}$$

In addition, the total income of households was calculated and then divided by the square root of the household size. At the denominator, the median income of the equated rural and urban households of the whole country as described above was placed against the urban and rural population of each province and used as the median income of the whole country. The individuals’ incomes were divided by the median income of the same region according to the living area (urban or rural) and finally, their mean was calculated.

$$\frac{\kern0.5em People\ with\ salary\ and\ wages\ and\ pension\ast 100\ }{Population\ over\ 65\ years\ of\ the\ province}=\mathrm{Salary}\ \mathrm{and}\ \mathrm{pension}\ \mathrm{coverage}$$

Accordingly, the total number of people having a pension and income from paying jobs was calculated and divided into the sampled total population in the province.

- Regarding the environmental empowerment dimension, the percentage of the elderly over 65 living in non-single households (independent living), the married elderly over the age of 65 (social communication), and the ratio of the households with the elderly over 65 (social communications) were used. In order to calculate all three indicators, the census data were used:
  $$\left( Number\ of\ households\ with\ over\ 65\ years\ old\ elderly\ supervisor\ in\ the\ province\right)/\left( Total\ households\ in\ the\ province\right)=\mathrm{Families}\ \mathrm{with}\ \mathrm{elderly}\ \mathrm{supervisor}$$

$$\frac{Married\ people\ over\ 65\ years\ old\ast 100\ }{Population\ over\ 65\ years\ of\ the\ province}=\mathrm{Percentage}\ \mathrm{of}\ \mathrm{married}\ \mathrm{people}\ \mathrm{over}\ 65$$

$$\left( People\ over\ 65\ are\ living\ in\ non- single- family\ households\ast 100\ \right)/\left( Population\ over\ 65\ years\ of\ the\ province\right)=\mathrm{Percentage}\ \mathrm{of}\ \mathrm{people}\ \mathrm{over}\ 65\ \mathrm{who}\ \mathrm{live}\ \mathrm{in}\ \mathrm{non}-\mathrm{single}-\mathrm{family}\ \mathrm{households}$$

- In the health status of the elderly, two indicators of life expectancy among 65-year-old men and women were used indicating their health:
  $$\mathrm{life}\ \mathrm{expectancy}\ \mathrm{of}\ \mathrm{men}\ \mathrm{and}\ \mathrm{women}\ \mathrm{of}\ \mathrm{every}\ \mathrm{province}-65=\mathrm{Life}\ \mathrm{expectancy}\ \mathrm{at}\ \mathrm{age}\ 65$$

After obtaining the life expectancy of individuals, this indicator was subtracted from 65 indicating the age of 65 years from life expectancy to reach life expectancy at the age of 65 years.

- Regarding the last dimension which is related to personal ability, which focus on the educational access of the elderly to educational centers as well as their access to the labor market, the percentages of the elderly over 65 with diploma and higher degrees (educational access), the percentage of the employed population over the age of 65 (the access of the elderly to the labor market), special economic participation rate of the elderly over 65, all of which are from the census data. The selected indicators are based on the theory given by Global Age watch. The method of measuring these indicators is as follows:
  $$\frac{Active\ population}{Total\ population}=\mathrm{Economic}\ \mathrm{Participation}\ \mathrm{Rate}\ \mathrm{of}\ \mathrm{special}\ \mathrm{age}$$

$$\frac{\ people\ over\ 65\ who\ is\ working\ast 100\ }{Population\ over\ 65\ years\ of\ the\ province}=\mathrm{percentage}\ \mathrm{of}\ \mathrm{people}\ \mathrm{over}\ 65\ \mathrm{who}\ \mathrm{is}\ \mathrm{working}$$

$$\left( Persons\ over\ 65\ years\ of\ age\ with\ a\ higher\ education\ than\ a\ diploma\ast 100\ \right)/\left( Population\ over\ 65\ years\ of\ the\ province\right)=\mathrm{Percentage}\ \mathrm{of}\ \mathrm{people}\ \mathrm{over}\ 65\ \mathrm{years}\ \mathrm{old}\ \mathrm{with}\ \mathrm{a}\ \mathrm{higher}\ \mathrm{education}\ \mathrm{than}\ \mathrm{a}\ \mathrm{diploma}$$

After calculating the raw indicators, the provinces were ranked using the TOPSIS technique according to the calculated indicators and the weighting of the indicators was performed by Shannon’s entropy method.

#### Shannon’s entropy method

In the TOPSIS technique, the Shannon’s entropy method is used. Knowing about the relative weights of the existing indicators is an effective step in the problem solving process. In Shannon’s entropy method, the decision matrix was formed at the first stage [27]. Then, the decision-making matrix scaling was performed according to the following equation:

$$Pij=\frac{R_{ij}}{\sum_{i=1}^mR_{ij}};j=1,n,\mathrm n\:\mathrm{number}\;\mathrm{of}\;\mathrm{indicators},\,\mathrm m:\:\mathrm{number}\;\mathrm{of}\;\mathrm{provinces}$$

Then, the weights of the indicators were calculated by calculating the j-th indicator and the degree of deviation (di) and the details of the relations at these steps are as follows:

$${\displaystyle \begin{array}{l} Measuring\ the\ the\ entropy\ of\ j- th\ indicator\\ {} E_{j}=-k\sum \limits_{i=1}^m\left[\mathrm{p_{ij}Lnp_{ij}}\right]\\ {}k=\frac{1}{Lnm}\\ {}=\mathrm{Index}\ \mathrm{weight}\kern1em {d}_j=1-{E}_j=\mathrm{Degree}\kern0.17em \mathrm{of}\kern0.17em \mathrm{deviation}\kern1em \mathrm{m}=\mathrm{Number}\ \mathrm{of}\ \mathrm{provice}\ \ Wj=\frac{d_{j}}{\sum_{j=1}^n d_{j}}\end{array}}$$

Then, the weight of the indicators was adjusted according to the importance of each dimension and the importance of indicators inside each dimension.

### Technique for order preference by similarity to ideal solution (TOPSIS)

TOPSIS technique is one of the ranking methods first proposed by Hawing and Yoon in 1981. This model is one of the best multi-index decision making models considering different weights for indicators. In this method, *m* options are evaluated by the *n* indicators. This technique is based on the notion that the selected option should have the shortest distance with a positive ideal solution and a maximum distance with an ideal negative solution. It was assumed that the utility of each indicator is uniformly incremental or decreasing [28]. The problem solving steps by the TOPSIS technique include decision-making matrix, transforming the decision matrix into an unscaled matrix, forming a weighted unscaled matrix (weighing method is described below), identifying the ideal positive and negative solution, calculating the distance between positive and negative options, and finally calculating the relative proximity of the indicator. The equation of the final step is as follows:

$$C_{i}=\frac{di^-}{{\mathrm d\mathrm i}^-+\mathrm d\mathrm i^+}$$

The final step in this method was ranking the provinces based on the obtained value. The greater value of this step represented a higher ranking for the relevant province. The most important limitation of this study was the inability to access all the required information for measuring all the indicators in active aging and Global Age Watch. For this reason, given the available data, only the above indicators could be calculated and described after calculating for all provinces and then the provinces were ranked.

## Results

After measuring 11 indicators related to health, well -being, and labor participation of the elderly, the results were indicated in Table 1.

**Table 1:** Indicators information

	Ability			Health status		Environmental empowerment			Income security		
Province	Economic Participation Rate of age above65(Access to the labor market)	Percentage of population over 65 who is working (ability of Elderly Employment)	Percentage of people over 65 years old with a higher education than a diploma(elderly Educational Access)	Life expectancy in men at the age of 65	Life expectancy in women at the age of 65	proportion of families with elderly supervisor above65 years old(social relations)	Percentage of married people over 65(social relations)	Percentage of people over 65 who live in non-single-family households(Relatives’ support)	Percentage of People with salary and wages and pension(Salary and pension coverage)	Average person’s income over 65 years into the average income of the rest of the population(Relative Welfare)	percentage of people with lower incomes than the median income of the country(elderly poverty rate)
Markazi	0.12	11.87	5.83	7.90	11.70	0.17	42.32	82.86	36.42	0.77	72.63
Gilan	0.15	13.89	11.21	7.10	11.50	0.18	41.44	80.82	43.32	0.91	68.26
Mazandaran	0.12	11.41	10.69	8.50	12.00	0.16	44.15	85.67	49.60	1.41	57.20
East Azarbayjan	0.14	14.08	7.81	7.50	10.90	0.16	44.08	87.30	39.71	1.28	64.06
West Azarbayjan	0.15	14.86	6.00	6.60	11.30	0.13	42.22	86.85	34.24	0.56	88.14
Kermanshah	0.15	14.50	6.12	5.40	10.30	0.16	43.30	88.33	34.78	0.73	80.58
Khozestan	0.12	11.84	7.26	6.30	8.90	0.11	43.87	93.75	60.85	0.80	75.80
Fars	0.14	13.88	11.21	7.10	11.30	0.15	44.99	87.90	40.34	0.77	75.91
Kerman	0.12	11.58	7.50	6.40	10.50	0.13	43.93	82.13	44.24	0.58	89.22
Khorasan razavi	0.12	12.24	9.30	7.10	11.20	0.14	43.80	73.91	33.88	0.64	84.30
Esfahan	0.09	8.68	11.44	8.00	12.00	0.16	45.25	75.13	48.25	0.66	81.35
Sistan and baluchestan	0.20	18.18	3.05	0.70	4.20	0.10	45.43	80.59	28.92	0.27	95.10
Kordestan	0.17	16.53	3.98	5.60	9.10	0.14	43.16	90.91	32.02	0.85	72.47
Hamedan	0.17	16.88	4.95	6.20	10.60	0.16	42.47	86.28	35.12	0.77	77.93
Chaharmahal and bakhtiari	0.13	12.03	2.81	5.20	10.00	0.15	45.56	80.27	45.58	1.18	70.80
Lorestan	0.15	14.51	4.59	6.10	9.50	0.15	45.49	87.97	42.98	0.62	85.96
Ilam	0.18	18.02	2.70	5.50	7.00	0.15	47.07	90.61	43.75	1.04	78.52
Kohgiluyeh and boyer ahmad	0.16	15.72	3.01	6.20	8.10	0.12	48.96	90.03	38.66	0.53	87.82
Bushehr	0.15	15.09	5.52	6.00	8.50	0.10	42.06	96.00	54.17	0.70	81.02
Zanjan	0.16	16.19	4.22	8.20	10.80	0.15	42.86	79.21	38.19	0.70	81.25
Semnan	0.08	7.53	10.14	8.20	11.10	0.16	43.83	79.71	64.64	1.02	64.26
Yazd	0.10	9.05	8.43	8.20	11.70	0.14	43.73	73.32	49.67	0.92	77.63
Hormozgan	0.18	17.91	3.56	4.90	8.70	0.10	43.01	89.61	46.28	0.61	83.88
Tehran	0.07	7.02	27.29	9.30	12.80	0.15	44.14	82.64	72.70	0.93	70.34
Ardebil	0.16	15.50	4.15	6.30	10.60	0.15	44.38	86.88	39.43	0.90	72.04
Qom	0.15	14.68	7.58	6.50	10.60	0.12	43.56	84.32	45.49	0.78	79.22
Qazvin	0.12	11.53	6.88	6.80	10.30	0.14	44.01	81.47	43.03	0.99	72.51
Golestan	0.16	16.06	7.11	5.80	9.20	0.12	41.95	83.47	43.30	1.16	79.04
North Khorasan	0.15	14.58	3.64	4.80	7.60	0.14	42.89	83.21	38.41	0.67	80.63
South Khorasan	0.13	13.25	4.14	5.80	8.90	0.18	44.29	65.50	25.23	0.58	88.30
Alborz	0.08	8.01	18.92	9.40	13.00	0.13	45.23	86.06	71.37	0.95	70.93

Source: research calculations

Based on the results obtained on the indicators in each province, the following findings were achieved. In the personal capability dimension of the elderly, economic participation rate among the people above 65 years old was the minimum in Tehran province while maximum in Sistan and Baluchestan province. The percentage of employed population aged 65+ indicating the access of the elderly to the labor market, this indicator was maximum in Sistan and Baluchestan province while minimum in Tehran province among the people above 65. The percentage of the elderly over 65 with a diploma and higher degree indicated that Tehran allocated the maximum value at the level of literacy and their access to education while Ilam province had the minimum value. Regarding the dimension of social and environmental empowerment, the proportion of the households with a householder over 65 years of age, which is an indicator of the social relationships, was the maximum in Guilan and South Khorasan provinces while it was minimum in Bushehr, Hormozgan, and Sistan and Baluchestan provinces. Another indicator indicating social communication is the percentage of the married elderly over 65, by which Kohgiluyeh and Boyer Ahmad province had the maximum value while Guilan province had the minimum value. In addition, the percentage of the elderly over 65 living in non-single households indicating the support of their relatives was maximum in Bushehr province while minimum in South Khorasan province. Regarding the health status dimension, the life expectancy of males and females over 65 was maximum in Alborz province while minimum in Sistan and Baluchestan province indicating their health status.

In the income security dimension, the pension and salary coverage indicator is the percentage of the people over 65 receiving salary which was maximum in Tehran province while minimum in South Khorasan province. In terms of relative welfare indicator, Mazandaran had the maximum while Sistan and Baluchestan had the minimum economic prosperity. In terms of aging poverty rate, Sistan and Baluchistan had the maximum economic poverty rate while Mazandaran had the minimum poverty rate.

After calculating the development thresholds (Table 2), Table 3 indicates the TOPSIS results on ranking the provinces based on the desired indicators.

**Table 2:** Classification thresholds for the level of development of active aging

Development level	Bottom limit	upper limit
Developed	0.619	0.909
Semi-developed	0.328	0.619
Less developed	0.037	0.328

**Table 3:** Results of TOPSIS on the development of the provinces in terms of active aging

Rank	Province	PI	
1	Tehran	0.9092	Developed
2	Alborz	0.6599	
3	Mazandaran	0.3871	Semi-developed
4	Gilan	0.3753	
5	Esfahan	0.3612	
6	Fars	0.3605	
7	Semnan	0.3503	
8	East Azarbayjan	0.2814	Less developed
9	Khorasan razavi	0.2760	
10	Yazd	0.2638	
11	Qom	0.2263	
12	Khozestan	0.2251	
13	Qazvin	0.2246	
14	Golestan	0.2194	
15	Kerman	0.2030	
16	Markazi	0.1933	
17	Kermanshah	0.1714	
18	Ardebil	0.1603	
19	Chaharmahal and bakhtiari	0.1572	
20	Bushehr	0.1566	
21	West Azarbayjan	0.1526	
22	Kordestan	0.1522	
23	Hamedan	0.1515	
24	Zanjan	0.1310	
25	Ilam	0.1258	
26	Lorestan	0.1148	
27	North Khorasan	0.1076	
28	Hormozgan	0.0974	
29	South Khorasan	0.0922	
30	Kohgiluyeh and boyer ahmad	0.0785	
31	Sistan and Baluchestan	0.0375	

The results of the provincial ranking indicated that only two provinces of Tehran and Alborz were well placed in terms of the development of the elderly, while about 77% of the provinces had no appropriate status for the elderly such as Sistan and Baluchestan and South Khorasan provinces.

In general, although some indicators such as personal capability were appropriate in some provinces such as Sistan and Baluchestan and South Khorasan, the indicators such as environmental empowerment, income security, and health status were not appropriate. In other words, the less developed provinces experienced low educational access, low social support, low social security, low income security, high poverty rate, and poor health status, despite the access of the elderly to the labor market. Such factors should be considered while planning regionally for the elderly. In order to rank the provinces using the TOPSIS technique, it was necessary to determine the weight of the indicators. At this stage, the most important indicator was the percentage of people over the age of 65 having diploma and higher academic degrees based on Shannon’s entropy while the lowest significant indicator was the percentage of the married elderly over 65 years old. Based on this technique, the indicator with the most difference between the provinces has more weight. Then, the results were determined by using the TOPSIS as shown in Table 4.

**Table 4:** Weights of Shannon entropy for the indicators used in TOPSIS

Rank	Indicators used	Wj
1	Percentage of people over 65 years old with a higher education than a diploma	0.4325
2	percentage of people with lower incomes than the median income of the country	0.1735
3	Average person’s income over 65 years into the average income of the rest of the population	0.1049
4	Life expectancy in men at the age of 65	0.0837
5	Percentage of People with salary and wages and pension	0.0726
6	Life expectancy in women at the age of 65	0.0391
7	Economic Participation Rate of age above65	0.0366
8	Percentage of population over 65 who is working	0.0362
9	proportion of families with elderly supervisor above65 years old	0.0128
10	Percentage of people over 65 who live in non-single-family households	0.0069
11	Percentage of married people over 65	0.0011

The results of Shannon’s entropy for weighting the indicators indicated that the three indicators allocating the maximum weight were the percentage of the people over 65 years old with diploma and higher degrees, percentage of the people with lower incomes than the median income, and average income of the people over 65 years old than the other people in society. The first factor indicates the elderly’s level of literacy and their educational access and is a very important factor in empowerment.

The second factor indicates the poverty rate of the elderly determining the number of people having no appropriate economic situation or access to the minimum standards. The third factor indicates the life and income security and the possibility of providing an independent life for them.

## Discussion

Based on the results obtained from the TOPSIS technique, all provinces were divided into three groups of developed, semi-developed, and less developed. Only two provinces of Tehran and Alborz were placed in the first category having more appropriate status than the other provinces. In addition, the provinces of Mazandaran, Gilan, Esfahan, Fars and Semnan were classified in the semi-developed provinces. All other provinces were placed in the less developed group. The results indicated that Tehran and Alborz provinces have the best status while Kohgiluyeh and Boyer Ahmad and Sistan and Baluchestan have the worst status.

Since the present study was conducted for the first time and no other study was available in this field, the results cannot be compared with other studies. However, based on the studies conducted on the non-elderly investigating only the economic development of the provinces of Iran, Khakpour and Davarinezhad [29] indicated that the provinces of Tehran, Alborz, Mazandaran, Khuzestan, Fars, Isfahan and Khorasan Razavi have the most economic development while the most deprived provinces included Sistan and Baluchestan, Kohgiluyeh and Boyer Ahmad, Chahar Mahal and Bakhtiari, Qom and Ilam which is consistent with the results of provincial ranking in this study. In addition, Pourasghar et al. [30] measured the development of the provinces of the country and determined that Tehran and Semnan provinces are the most developed provinces while Kurdistan, Sistan and Baluchestan, and Hamedan have the least development in the country, which is largely inconsistent with the results of the present study. The results of this study indicated that the elderly of Sistan and Baluchestan province are more involved in economic participation and the number of employed people allocates a higher contribution than all other provinces. However, the median income of their elderly people is the lowest in comparison to the median income of the rest of the population suggesting that the income of the elderly in this province is not sufficient and income is less than the elderly of other provinces although they are working, leading to an increase in the poverty rate. Sistan and Baluchestan province has the highest share in the elderly poverty rate, which is considered as a negative factor. Due to the high participation rate of the elderly in Sistan and Baluchistan in the labor market, a limited number of them had access to educational services indicating that this province has the lowest number of the educated elderly after Ilam. As Arpino et al. believe that policies devoted at stimulating an active participation in society among older people should be particularly focused on lower educated groups [31]. Therefore, all these findings indicate the lack of economic and social development of this province. On the other hand, Tehran province, which is in the best development for the elderly, is one of the provinces with the lowest rates in terms of economic participation and the elderly in this province receive more appropriate income than the rest of the population which can be related to other miscellaneous incomes.

In addition, Hosseini [32] studied the relationship between mortality and development in Iranian provinces and indicated a direct and significant relationship between life expectancy at birth and development in the provinces of Iran. Further, it was found that the lowest rate of life expectancy is in Sistan and Baluchestan province while the maximum rate of life expectancy is related to the provinces of Tehran, Gilan, Isfahan, Semnan, Qom, and Fars. In terms of the degree of development, the provinces of Sistan and Baluchestan and Kurdestan have the lowest life expectancy rate while the provinces of Tehran, Isfahan, Semnan, and Yazd have the highest life expectancy rate. This study indicated that Sistan and Baluchestan province has the lowest level of life expectancy and development in the elderly which can be due to poor economic and social conditions in the province. Furthermore, the elderly people are forced into economic activity at an older age due to the lack of income than other provinces, which can increase their physical and mental health and reduce or endanger their health. Additionally, after Alborz province, Tehran have the highest life expectancy in men and women and the participation rate of the elderly in economic activities in these provinces is much lower than other provinces while the median income of the elderly in Tehran is in an appropriate condition, compared to other people of the country. Such factors confirm the better economic and social conditions in Tehran province than Sistan and Baluchistan improving the health conditions of the elderly and increasing their life expectancy. As a result, the better economic and social conditions of the provinces lead to less economic activity and less pressure among the elderly resulting in their improved health conditions. In addition, higher health leads to the reduction of health expenditures among the elderly and improvement of their economic conditions.

Furthermore, the conditions for access to the labor market and high income, as well as the acquisition of education, were facilitated due to the more construction, development, and welfare activities and educational facilities in some provinces of the country such as Tehran and Alborz. More developed facilities and health services available in these provinces lead to the improvement of health conditions and ultimately the living conditions of the elderly in these provinces, which results in developing these provinces.

## Conclusions

In the present study, four indicators had the highest importance included the percentage of the elderly with a diploma and academic degree, the percentage of the elderly with lower incomes than the median income, the median income of the elderly to the median income of the other people in society, and life expectancy among the 65-year old men. As a conclusion, the government measures and policies were highly effective in improving the economic, social and health conditions of the provinces. Thus, the rate of growth and development in deprived provinces should be increased and the indicators having the lowest levels should be identified in order to plan appropriately. In other words, personal and social empowerment should be facilitated, some measures should be taken for improving the miscellaneous income of people, and health facilities should be provided to the elderly to reduce their health cost burden and improve the state of active aging in less developed provinces.

## Data Availability

The raw dataset used for the technical analyses in the present study is publicly available online at: 1. https://www.amar.org.ir/english

## Abbreviations

WHO: World Health Organization
TOPSIS: Technique for order preference by similarity to ideal solution
